# Equitable Patient and Public Involvement Strategies for Scaling Health and Social Care Interventions: A Consensus Study Inspired by Delphi Methods

**DOI:** 10.1111/hex.70850

**Published:** 2026-09-09

**Authors:** Roberta de Carvalho Corôa, Odilon Quentin Assan, Ali Ben Charif, Amédé Gogovor, Kathy Kastner, Robert K. D. McLean, Andrew Milat, France Légaré, The RePOS Network

**Affiliations:** ^1^ Faculty of Medicine Université Laval Quebec Canada; ^2^ VITAM – Centre de recherche en santé durable Centre intégré universitaire de santé et services sociaux de la Capitale‐Nationale Quebec Canada; ^3^ Unité de soutien au système de santé apprenant Québec Quebec Canada; ^4^ CubecXpert Quebec Canada; ^5^ Best Endings Toronto Canada; ^6^ International Development Research Centre Ottawa Canada; ^7^ School of Public Health University of Sydney Sydney Australia; ^8^ New South Wales Agency for Clinical Innovation St. Leonards Australia

**Keywords:** co‐construction, health and social services, implementation science, participatory research, patient and public engagement, scalability, scaling science

## Abstract

**Background:**

Patient and public involvement (PPI) is recognized as essential for scaling interventions in health and social services, but there are few guidelines to support it. We sought consensus on strategies for involving patients and the public in scaling in an equitable and meaningful way.

**Methods:**

We conducted a participatory consensus study inspired by Delphi techniques. We used as a starting point 23 PPI strategies for scaling (on the involvement continuum, from providing information to full co‐production) as identified in a 2024 scoping review. First, we established a steering committee, intended to include patient or public representatives, to validate the relevance of the 23 strategies for inclusion in an online survey consensus process to confirm their applicability to PPI. Second, researchers, health professionals, policymakers, and patient or public representatives completed the online survey to assess the validated strategies against four evaluation criteria: equity, comprehensiveness, adaptability and significance. We used the DELPHISTAR checklist to report results and the GRIPP2 framework to report PPI in the study.

**Findings:**

All 23 PPI strategies were found relevant by the steering committee and proceeded to the 2‐round consensus process to evaluate 92 items (23 strategies x 4 criteria). By April, 2025, 14 participants had completed the first round and nine had also completed the second. Participants included patient and public representatives (*n* = 5), researchers (*n* = 6), health professionals (*n* = 3) and policymakers (*n* = 2) mostly based in Canada (*n* = 10). Of 23 PPI strategies assessed, 19 achieved consensus on at least one of the four criteria. Three strategies (Service users' needs assessment, Organizational advisory groups and Co‐leadership in quality and safety improvement) achieved consensus across all criteria, while four strategies did not reach consensus on any. Co‐leadership in quality and safety improvement (88.3%), Co‐leadership in policymaking (87.3%) and Service users' needs assessment (87.3%) achieved the highest global scores. Consensus was reached on 44/92 items, most frequently for equity. These strategies were primarily situated in the organization of health and social care and the co‐production level of the involvement continuum.

**Conclusions:**

The strategies prioritized by stakeholders were not necessarily the ones most frequently discussed in the literature. We recommend systematically involving patient and public representatives throughout scaling, including when choosing PPI strategies.

**Patient or Public Contribution:**

A patient instructor was involved in the steering committee, sharing decisions on study design, data collection instruments and dissemination of findings. Patient and public representatives also took part in the consensus process, and their feedback was considered equal to that of the other experts.

## 1 Background

The scaling of health and social care interventions is a systematic, evidence‐informed process aimed at increasing the intended impacts of interventions that have proven effective [1]. This can be achieved by expanding coverage, replicating an intervention that has proven effective across further settings, or improving its quality while maintaining effectiveness, thereby enabling more people to benefit from it [2, 3]. For example, efforts to scale up the global COVID‐19 vaccination program included expanding vaccine manufacturing, establishing regional production hubs through technology transfer and strengthening distribution systems to improve equitable access while maintaining vaccine quality [4]. Recently, discussions have grown regarding the importance of considering equity and ethical principles in scaling [2, 5, 6, 7, 8]. Specifically, equity has emerged as a priority, reflecting the Quintuple Aim for healthcare improvement which emphasizes that all improvement and innovation efforts should focus on individuals and communities with the greatest needs [9]. The goal is to ensure that everyone has a fair opportunity to attain their full health potential and that no one is disadvantaged in achieving it [10]. In the context of scaling healthcare interventions, equity means shifting the focus from simply reaching maximum scale to maximizing impact by specifically engaging disadvantaged communities throughout the scaling process [11, 12].

Patient and public involvement (PPI) in scaling processes is therefore crucial. From a social justice perspective, Fraser's theory of participatory parity argues that justice requires all people to have genuine opportunities to participate as equals in decisions that affect their lives [13]. Meaningful PPI can contribute to this goal by redistributing decision‐making power, recognizing experiential knowledge alongside professional expertise, and creating opportunities for groups who have historically been excluded from health decision‐making to influence policies, services and implementation processes [14, 15]. However, PPI literature has paid limited attention to equity [16]. PPI practices often overlook people living in marginalized conditions and fail to account for how power imbalances, privilege, discrimination, financial inequities and inaccessible communication shape opportunities for meaningful participation and involvement in healthcare [15, 16].

In scaling, PPI should ensure that the perspectives of those who should be the primary beneficiaries of the scaled health and social care interventions are represented. Involving people from diverse backgrounds from the beginning of the scaling process should also help ensure that scaling does not exacerbate health inequalities or cause harm to certain groups [6]. Nonetheless, a 2017 systematic review on scaling strategies found a lack of PPI in scaling, which could suggest a key barrier to effectively scaling health and social interventions [17]. Scaling processes tend to be top‐down initiatives, driven by policymakers and researchers, that do not take into account of the values, preferences and perspectives of intended beneficiaries [17, 18]. To address this gap, a scoping review was conducted which identified and characterized 23 PPI strategies reported in the literature for use in scaling [8, 19]. This scoping review confirmed that PPI in scaling can generate positive outcomes for both those involved and for healthcare systems, and that involvement can occur across all phases of scaling (e.g., planning, implementation and evaluation). In tools for scalability assessment, an essential preliminary step in scaling, PPI in scaling has been recognized as a key mechanism for advancing equity [2, 5, 20].

While PPI in scaling is now widely recognized as essential, qualitative evidence drawn from experience remains scarce [8]. There is a need for evidence‐informed and practical guidance to ensure that involvement is not only present but also meaningful and equitable. This should go beyond tokenistic participation, i.e. the input of patient and public representatives should carry equal weight to that of policymakers and researchers. Policymakers and researchers also need to address the multiple barriers in involving lay participants in this expert‐dominated domain. These barriers include the exclusionary use of jargon and technical language, as well as the nature of scaling decisions, which are often made at the highest levels of healthcare systems and political institutions [8].

We therefore aimed to identify and prioritize strategies for meaningfully involving patients and the public in scaling health and social care interventions through a consensus process among diverse scaling stakeholders. By “meaningful involvement,” we mean engagement that is (1) equitable (has the potential to involve people from diverse backgrounds), (2) comprehensive (involves people in all the phases of scaling), (3) adaptable (to diverse levels of care or sociocultural contexts) and (4) significant (not tokenistic). These criteria are important not only for increasing scaling effectiveness but also for generating outcomes relevant to those involved. This work offers insights into strategies to support people‐centered scaling.

## 2 Methods

### 2.1 Study Design and Overview

We conducted a participatory consensus study using Delphi techniques. Following the principles of participatory research, we aimed to engage stakeholders to work alongside the research team throughout all stages of the research process [21]. First, to ensure project governance and a participatory planning process, we established a steering committee. Second, inspired by the RAND/UCLA Appropriateness Method, a Delphi technique, we assessed strategies for meaningfully involving patients and the public in scaling health and social care interventions [22, 23]. Delphi processes bring together diverse stakeholders to reach consensus on health‐related priorities and, by allowing participants to remain anonymous, are particularly useful for reducing power imbalances among stakeholders from different backgrounds [21, 24]. In our consensus process, the steering committee selected strategies deemed relevant from an initial list of 23 PPI strategies for scaling health and social care interventions identified in a 2024 scoping review [8]. We conducted a two‐round online consultation with diverse scaling stakeholders, including patient and public representatives, to assess selected strategies against four criteria: equity, comprehensiveness, adaptability and significance. These criteria were selected because they respond to recommendations in the literature calling for (a) greater attention to equity in both scaling and PPI [6, 7, 12, 16, 25]; (b) early involvement of those concerned in the scaling process [8, 26]; (c) recognition of the central role of adaptability in scaling [20], and (d) the avoidance of tokenism when involving patients and the public in healthcare, policymaking and research [15]. Therefore, they reflect the capacity of a PPI strategy to equally involve different groups, across various scaling phases and sociocultural contexts, and in a meaningful way [8, 19].

We considered a PPI strategy in scaling as any method aimed at enabling patient and public representatives to inform, advise, collaborate in or co‐construct the scaling process.

**Figure 1:**
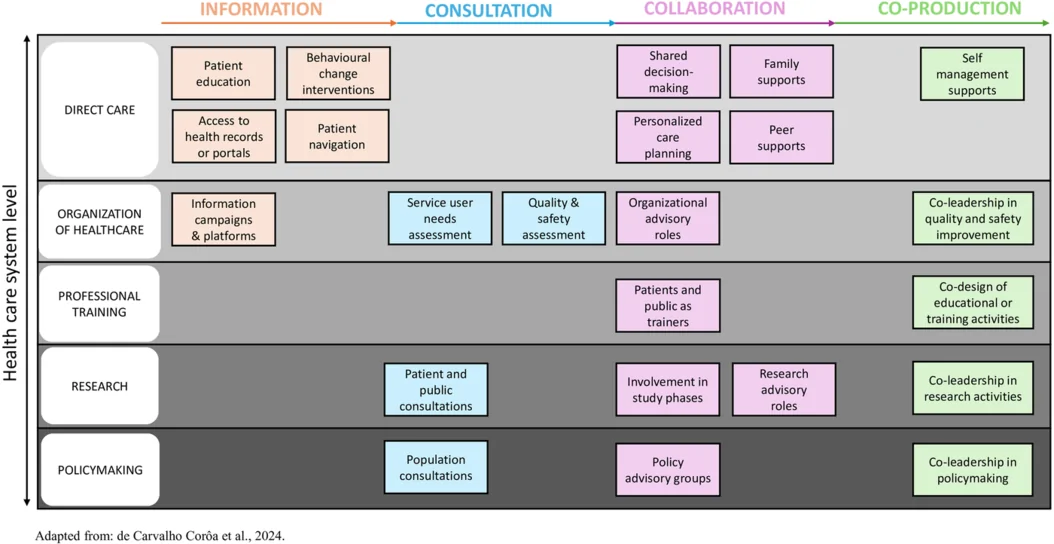
Mapping of PPI strategies across healthcare system strata and levels of the involvement continuum.

PPI strategies in scaling are situated along a continuum of levels of involvement, ranging from sharing information to full co‐production, and can be applied at any stratum of the healthcare system (from direct care to policymaking) in which the scaling is to occur (Figure 1) [8, 27, 28, 29]. Additional details on the strategies and their definitions can be found in the published scoping review by de Carvalho Corôa et al. [8].

This study was led by an interdisciplinary team of researchers in the fields of medicine (France Légaré), sociology (Roberta de Carvalho Corôa) and epidemiology (Odilon Quentin Assan), in collaboration with experts from diverse backgrounds (e.g., policymaking, clinical practice, and trainees), as well as a patient representative. In developing the study, we first followed the integrated Knowledge Translation (iKT) approach by establishing a steering committee that included a patient representative to foster collaboration and provide feedback on the research process [30, 31]. Subsequently, we applied the RAND/UCLA Appropriateness Method adapted to reach a consensus on the most promising PPI strategies.

We reported PPI in our study using the GRIPP2 checklist and prepared this article in accordance with the DELPHISTAR reporting guideline [32, 33]. The protocol was published in a peer‐reviewed journal and is available online [19].

### 2.2 Ethics Approval

We obtained ethical approval for this project from the Ethics Board of the Centre Intégré Universitaire de Santé et de Services Sociaux (CIUSSS) de la Capitale‐Nationale (2021–2019). Electronic and written informed consent was obtained from all the participants via the REDCap platform [34].

### 2.3 Steering Committee Composition and Feedback

Following the Practical Guide for researchers working in Scaling Science, an evidence‐based, action‐oriented tool, we aimed to involve a wide range of stakeholders across the scaling spectrum in our study [26]. As recommended by the guide, these stakeholders included initiators (e.g., innovators and researchers), enablers (e.g., policymakers and service providers) and people affected by scaling (e.g., patients, members of the public and communities). We then established a steering committee composed of stakeholders with expertise or lived experience relevant to the scaling of health and social service innovations. The primary objectives of the committee were to provide external validation of our study methods and to review and refine the 23 PPI strategies to be assessed in the consensus process. By involving a diverse group of potential end‐users of PPI strategies, we sought to ensure methodological rigour, stakeholder relevance and practical applicability of the study results.

#### 2.3.1 Participants, Eligibility Criteria and Recruitment

For the committee, we aimed to recruit at least two academic researchers and two policymakers with recognized expertise in scaling health or social care interventions, two practicing health professionals and one patient and/or public representative (e.g., a patient partner or caregiver). These minimum recruitment targets were established to ensure that all key stakeholder groups were represented on the committee. A minimum of two researchers, two policymakers and two practicing health professionals were sought to capture variation in professional roles and perspectives within each stakeholder group. The aim of including patient and/or public representatives was to ensure that their experiential knowledge as healthcare system users was reflected in the committee and one representative was deemed adequate to represent this perspective. Researchers and policymakers were required to have expertise in scaling health or social care innovations across any type of intervention or delivery context. Practicing health professionals were not required to have experience in scaling but were expected to have an interest in the subject. The patient and/or public representative represented the perspective of a healthcare system user rather than a specific health condition, as the study focused on scaling health and social care interventions across diverse contexts. To be eligible for the steering committee, participants had to: (a) be ≥ 18 years of age; (b) be able to actively participate in committee activities (online or in writing); (c) be able to read and understand English or French; and (d) demonstrate knowledge of, or interest in, the scaling of innovations in health and social services.

Recruitment was conducted purposively. We invited co‐authors of a published scoping review on PPI strategies in scaling initiatives [8] as well as scaling experts and collaborators within our professional networks. Invitations were sent by email and included information on study objectives, expectations of committee members and anticipated time commitment.

#### 2.3.2 Data Collection and Analysis

We sought both general and targeted feedback from steering committee members. First, we asked them to review and comment on the overall study methods. Second, we requested specific input on the validation of the 23 PPI strategies to be assessed later in the consensus process. To facilitate this, we distributed an online survey that included both structured and open‐ended components. For each PPI strategy, committee members were asked to evaluate: whether the strategy adequately represented the construct of patient and public involvement, that is, whether it reflected the concept and practice of involving patients and the public in health and social care as holders of valuable experience and knowledge; and whether the strategy explicitly targeted patients and/or the public, that is, whether it was not exclusively directed toward other groups, such as policymakers. Responses were recorded on a 5‐point Likert scale ranging from 1 (“strongly disagree”) to 5 (“strongly agree”). Open‐ended fields were provided for members to elaborate on their ratings, offer alternative wording and suggest additional strategies, but completion was optional. PPI strategies for which more than 80% of the steering committee members provided ratings of 3, 4 or 5 on the two evaluation questions were retained for inclusion in the subsequent consensus phase. Quantitative data from Likert‐scale responses were summarized using descriptive statistics (frequency distributions). Qualitative feedback from open‐ended responses was limited in volume; therefore, formal coding was not required. Instead, comments were reviewed manually and analyzed to identify specific suggestions and areas for refinement. All results were synthesized into summary tables using Microsoft Excel. The synthesized results were intended to guide future refinements of the proposed PPI strategies and adjustments to the design of the consensus process (e.g., to refine the wording of the questions).

### 2.4 The Consensus Process

The consensus process was adapted from Delphi techniques, which are widely recommended for obtaining expert consensus in contexts where evidence remains limited or inconclusive [23, 35]. We chose to proceed with an online asynchronous consultation, maintaining participant anonymity to minimize power imbalances and to enable individuals from diverse backgrounds (e.g., patients and/or public representatives, policymakers) to express their perspectives freely and without undue influence [21, 24]. Our consensus process was conducted in two rounds using the REDCap platform in both French and English.

#### 2.4.1 Participants, Eligibility Criteria and Recruitment

Based on sampling trends and recommendations from previous Delphi studies, we intended to recruit a minimum of 16 scaling stakeholders to complete both rounds of the consensus study [35, 36]. Our goal was to include at least four patients or public representatives, two health professionals, two policymakers, two trainees, two members of patient‐oriented research support institutions (e.g., members of Support for People and Patient‐Oriented Research and Trials in Canada) and four scaling researchers. Support for People and Patient‐Oriented Research are regional organizations that promote patient‐oriented research by offering specialized services to researchers, patients, clinicians and decision makers. They contribute to advancing equity and patient engagement across Canada. Participants were eligible if they were at least 18 years old, able to provide informed consent, able to actively participate and read and understand English or French and had knowledge of or interest in scaling health and social care interventions. We used two recruitment strategies. First, we emailed authors of studies included in reviews on scaling conducted by our team [17, 37]. Second, we contacted potential participants from our professional network who had previously collaborated with us on scaling studies [1, 8, 20]. No compensation was offered for participation.

#### 2.4.2 Data Collection

##### 2.4.2.1 Data Collection Instruments

We collected data for the consensus process via the REDCap platform using a sociodemographic questionnaire and a survey on PPI strategies in scaling. The sociodemographic questionnaire included questions on participants' country, gender, ethnic/racial identification, scaling role (e.g., policymaker, public representative), organization (e.g., academic, government) and years of practice in scaling. This was based on variables recommended by Statistics Canada. To ensure their suitability for an international panel, the response options, including those for ethnicity, were reviewed and refined by our multicultural research team and steering committee [38]. In the survey, we provided definitions of each PPI strategy included for assessment and asked participants to rate it using a 5‐point Likert score according to whether it has potential for equity [39]; for comprehensiveness [40]; for adaptability [20]; or for significance [41]. Definitions of evaluation criteria are presented in Box 1.

Box 1 **Definitions of the four evaluation criteria.**

**Table:** 

**Equity:** Refers to the potential of the strategy to involve patient and public representatives from diverse ethnic, sex, gender, health and socioeconomic backgrounds [39].
**Comprehensiveness:** Refers to the potential of the strategy to be applied across all phases of scaling, including scalability assessment, development of a scaling plan, preparation for scaling, implementation and evaluation/monitoring [40].
**Adaptability:** Refers to the possibility of applying the strategy across different contexts, such as countries with varying income levels, and at primary, secondary and tertiary levels of care [20].
**Significance:** Refers to the extent to which the strategy fosters the involvement of patients and the public as equal members of the scaling team, ensuring genuine rather than tokenistic participation [41].

##### 2.4.2.2 Consensus Process

The consensus process was conducted in two rounds. First, participants received the link to access the REDCap platform and signed the consent form. Once consent was provided, they were anonymized within the platform and identified only by a code number. Second, when participants from all the expected categories had signed the consent form, the research team sent a second link to complete a sociodemographic questionnaire and a survey designed to assess the PPI strategies deemed relevant by the steering committee for scaling. This consisted of 92 evaluation items, representing each retained strategy (up to 23) multiplied by the four evaluation criteria [4]. Third, participants who completed the first survey were invited to complete a second one in which they were shown the consensus median score for each strategy that had not reached the required level of agreement. They were then asked to re‐assess these strategies, either confirming or revising their evaluations using the same Likert‐type scales. In both rounds, the surveys included open‐ended questions to collect comments and suggestions and to allow participants to justify their responses. However, completion of open‐ended questions was optional. After each survey was distributed, automatic reminders were sent via the REDCap platform 7 days later and continued until the survey completion period was closed. The research team monitored survey completion as closely as possible to ensure that all expected participant categories completed the process, and both surveys were closed once the project deliverable deadlines had been reached.

#### 2.4.3 Data Analysis

Frequencies were calculated and descriptive analyses of sociodemographic data were performed using SAS 9.4 software to obtain the median score and percentage of agreement [42]. In the first round, consensus was defined as ≥ 75% agreement on priority scores of 4–5 for equity, comprehensiveness, adaptability and significance. In the second round, consensus was considered reached when ≥ 80% of participants rated a strategy 4–5. The threshold of the first round was intentionally set lower to promote inclusivity, ensure that potentially relevant strategies were not prematurely discarded, and allow participants to reflect on a wide range of perspectives before narrowing down [22]. The higher threshold of the second round was adopted to strengthen the robustness of findings and reduce uncertainty [43]. Therefore, the first round served as a filtering and learning phase, while the second ensured methodological rigour and a strong consensus on the final outcomes [43]. Qualitative data were limited, and a formal thematic analysis was not required. However, the data were organized in an Excel spreadsheet and discussed by the team. We used quotations to illustrate participants' perspectives rather than using the frequency of comments.

### 2.5 Deviations From Protocol

The consensus process remained largely consistent with the original protocol [19]. However, as mentioned above, we adjusted the consensus threshold in the first round to 75% to promote inclusivity and allow participants to familiarize themselves with the process before retaining only the items that reflected the strongest and most stable agreement. In addition, although the protocol initially planned three rounds, we performed only two, as a third round was deemed unnecessary. Finally, we used a 5‐point Likert scale rather than a 10‐point scale to minimize respondent burden and reduce complexity for patient and public participants, while still capturing sufficient variation in ratings to distinguish levels of agreement.

## 3 Results

We report the main results from the steering committee's contributions and the assessment of PPI strategies during the consensus process. Qualitative data are reported to enrich and complement the main descriptive findings. The steering committee was recruited and their input collected between December 2023 and January 2024. The first round of the consensus process took place between September 2024 and February 2025, followed by the second round between March and April 2025.

### 3.1 Contribution of the Steering Committee

A total of 11 experts of the 13 invited accepted to take part in the steering committee. They represented a diverse range of stakeholders involved in scaling initiatives in health and social care. One was a patient instructor, one was a clinician, two were both policymakers and researchers, six were researchers and one was a trainee (PhD candidate whose thesis explores the concept of scaling). They were based in Canada (*n* = 8), both in Canada and Australia (*n* = 2) and in Brazil (*n* = 1). All 23 proposed PPI strategies met the threshold (≥ 80% of members rating 3, 4 or 5 on both evaluation questions) and were therefore retained for inclusion in the consensus process. Comments from steering committee members indicated that some PPI strategies (e.g., patient and public education) were passive forms of involvement. Participants also emphasized that no single strategy alone can ensure meaningful participation, which requires additional commitment and supportive resources such as guiding frameworks and tools.

### 3.2 Participant Characteristics

A total of 112 participants were invited to take part in the consensus process, of whom 23 signed the consent form. A total of 17 participants completed the sociodemographic questionnaire. Fourteen participants completed the first round, and nine of them also completed the second round (recruitment was two participants below the projected target). Two members of the steering committee were also included in the Delphi panel in their respective participant categories, one as a researcher with expertise in scaling and the other as a health professional. In the first round, gender distribution was equal. Participants were from Canada (*n* = 10), France (*n* = 2), Brazil (*n* = 1) and the United Kingdom (*n* = 1), and most reported French as their primary language (*n* = 7). They represented various sectors, with the largest proportion affiliated with academia (*n* = 6), followed by government institutions (*n* = 3). In terms of ethnic and racial self‐identification, most participants identified as White (*n* = 9), followed by Black (*n* = 3). Their roles in scaling initiatives mostly included researchers (*n* = 6), patients or public representatives (*n* = 5), health professionals (*n* = 3) and members of a patient‐oriented research support institution (*n* = 3).

**Figure 2:**
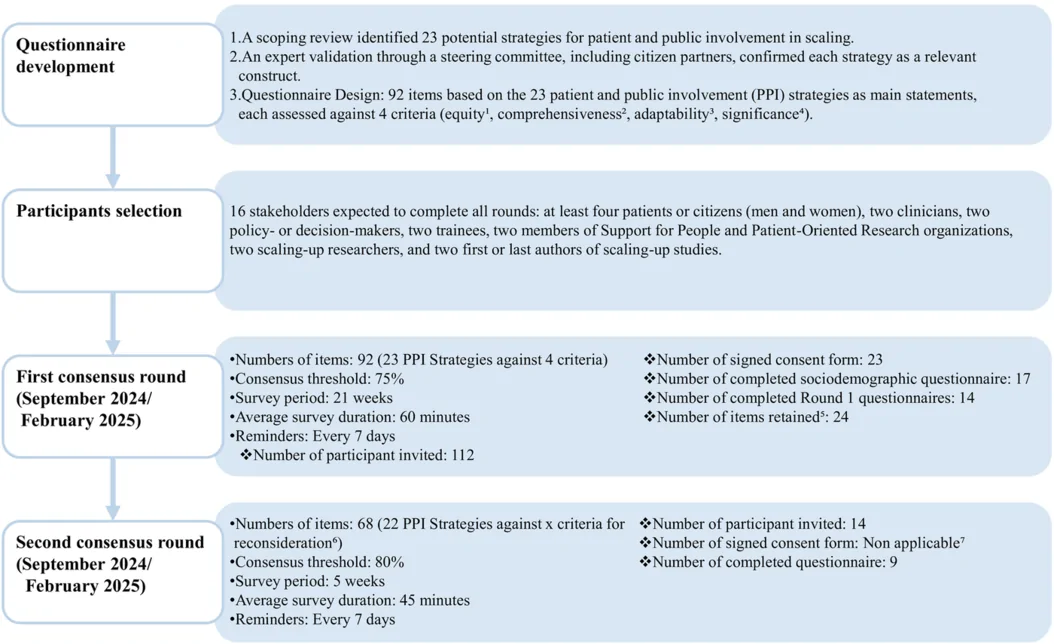
Flowchart and timeline of the participatory consensus process. **^1^Items retained:** PPI strategies and their associated criteria that reached ≥75% agreement during Round 1 were considered to have achieved consensus. ^2^ **For reconsideration:** PPI strategy that did not reach a minimum of 75% agreement (consensus) on one or more criteria during Round 1 were reconsidered for reassessment in Round 2. ^3^ **Non applicable:** Only participants who completed Round 1—that is, those who signed the consent form—were invited to participate in Round 2.

Participants who did not complete the second round identified themselves as researchers (*n* = 4), health professionals (*n* = 1) and policymakers (*n* = 1). They were mostly women (*n* = 3), from Canada (*n* = 4), identified as White (*n* = 3), and reported French as their first language (*n* = 3). The consensus process recruitment and participation flowchart is summarized in Figure 2. Participants characteristics are detailed in Table 1.

**Table 1:** Sociodemographic characteristics of participants who completed the sociodemographic questionnaire and participated in each consensus round.

	Completed sociodemographic questionnaire (*N* = 17)^a^, *n* (%)	Round 1 (*N* = 14), *n* (%)	Round 2 (*N* = 9), *n* (%)
**Age**			
25–34 years	3 (17.6)	3 (21.4)	3 (33.3)
35–44 years	2 (11.8)	2 (14.3)	0 (0)
45–54 years	5 (29.4)	5 (35.7)	2 (22.2)
55–64 years	1 (5.9)	0 (0)	0 (0)
65–74 years	4 (23.5)	3 (21.4)	3 (33.3)
75 years or more	2 (11.8)	1 (7.0)	1 (11.2)
**Gender**			
Male	8 (47.1)	7 (50)	5 (55.6)
Female	8 (47.1)	7 (50)	4 (44.4)
Prefer not to answer	1 (5.8)	0 (0)	0 (0)
**Country**			
Canada	11 (64.6)	10 (71.5)	6 (66.7)
France	2 (11.8)	2 (14.3)	2 (22.2)
Brazil	1 (5.9)	1 (7.1)	1 (11.1)
Mexico	1 (5.9)	0 (0)	0 (0)
UK	1 (5.9)	1 (7.1)	0 (0)
USA	1 (5.9)	0 (0)	0 (0)
**Province (Canada)**			
Quebec	10 (58.8)	9 (64.3)	6 (66.7)
Ontario	1 (5.9)	1 (7.1)	0 (0)
Other countries	5 (29.4)	4 (28.6)	3 (33.3)
Prefer not to answer	1 (5.9)	0 (0)	0 (0)
**Place of living**			
Large urban population centres^b^	12 (70.5)	10 (71.4)	5 (55.6)
Medium population centres^c^	1 (5.9)	1 (7.2)	1 (11.1)
Small population centres^d^	2 (11.8)	1 (7.2)	2 (22.2)
Rural area^e^	2 (11.8)	2 (14.2)	1 (11.2)
**Ethnic and racial self‐identification**			
Indigenous	0 (0)	0 (0)	0 (0)
White	10 (58.8)	9 (64.3)	6 (66.7)
Black	3 (17.6)	3 (21.4)	2 (22.2)
Latin American	1 (5.9)	1 (7.1)	1 (11.1)
Asian	0 (0)	0 (0)	0 (0)
Mixed	1 (5.9)	0 (0)	0 (0)
African	1 (5.9)	1 (7.1)	0 (0)
Prefer not to answer	1 (5.9)	0 (0)	0 (0)
**First language**			
French	8 (47.1)	7 (50)	4 (44.4)
English	3 (17.6)	2 (14.3)	1 (11.2)
Neither English nor French	6 (35.3)	5 (35.7)	4 (44.4)
**Scaling role** ^f^ **(totals represent roles selected, not participants)**			
Total roles selected^f^	25 (100)	20 (100)	14 (100)
Health professional	4 (16)	3 (15)	2 (14.3)
Policy maker	2 (8)	2 (10)	1 (7.1)
Trainee	1 (4)	1 (5)	1 (7.1)
Researcher	8 (32)	6 (30)	2 (14.3)
Patient or citizen partner	6 (24)	5 (25)	5 (35.7)
Member of a patient‐oriented research support institution	3 (12)	3 (15)	3 (21.5)
Prefer not to answer	1 (4)	0 (0)	0 (0)
**Social or employment role**			
Academic	7 (41.2)	6 (43)	3 (33.4)
Government	2 (11.7)	2 (14.3)	2 (22.2)
Retired	3 (17.6)	2 (14.3)	2 (22.2)
Industry	1 (5.9)	1 (7.1)	0 (0)
Government and GMF clinic	1 (5.9)	1 (7.1)	0 (0)
Charity	1 (5.9)	1 (7.1)	1 (11.1)
Health and development NGO	1 (5.9)	1 (7.1)	1 (11.1)
Health System Research Institute	1 (5.9)	0 (0)	0 (0)
**Years of experience**			
1–5 years	6 (35.3)	6 (42.8)	5 (55.6)
None	1 (5.9)	0 (0)	0 (0)
Less than 1 year	2 (11.8)	2 (14.3)	1 (11.1)
6–14 years	2 (11.8)	2 (14.3)	0 (0)
15 years or more	6 (35.2)	4 (28.6)	3 (33.3)

^a^ Twenty‐three individuals provided consent to participate in the study; however, only 17 completed the sociodemographic questionnaire and were therefore included in this table.

^b^ Large urban population centres (consisting of a population of 100,000 and over).

^c^ Medium population centres (with a population of between 30,000 and 99,999).

^d^ Small population centres (with a population of between 1000 and 29,999).

^e^ Rural area (rural areas include all territory lying outside population centres).

^f^ Scaling role was a non‐mutually exclusive characteristic. Participants could select more than one role; therefore, totals represent the number of roles selected rather than the number of participants.

### 3.3 Assessment of PPI Strategies in Scaling

#### 3.3.1 Consensus Overview

All 23 PPI strategies in scaling were evaluated against the four criteria of equity, comprehensiveness, adaptability and significance through the consensus process. In the first round, 92 item assessments were made (four criteria for each of the 23 strategies). Consensus was reached for 24 items, and only one PPI strategy achieved consensus across all four criteria. In the second round, 68 items were assessed, of which 20 reached consensus, and two PPI strategies achieved consensus across all four criteria. Overall, 44 items reached consensus and three PPI strategies achieved consensus across all four criteria.

#### 3.3.2 First Round

Across all strategies assessed in the first round, the mean consensus score was 64.4% (SD = 13.2%), with a median of 64.3% (range: 30.8%–92.9%). Thirteen PPI strategies reached the predefined consensus threshold of ≥ 75% on at least one of the four assessment criteria. Only one strategy, Organizational advisory groups (Organization of health and social care level), reached consensus across all four criteria (equity 76.9%, comprehensiveness 85.7%, adaptability 78.6%, significance 78.6%). Among the remaining strategies, Co‐leadership in quality and safety improvement (Organization of health and social care level) achieved the highest overall consensus score, with scores of 85.7% for equity, 85.7% for comprehensiveness and 92.9% for significance. The next was Patient and public education (Direct care level), which reached consensus on three criteria with scores of 76.9% for equity, 75.0% for comprehensiveness and 76.9% for adaptability.

Of the 23 PPI strategies assessed, nine achieved consensus for equity, six for comprehensiveness, two for adaptability and seven for significance. Ten strategies did not reach consensus on any of the four criteria: personalized care planning, self‐management supports, shared decision making and access to health records or portals (Direct care level); information campaigns and platforms and quality and safety assessment (Organization of health and social care level); Patients and public as trainers (Professional training level); Patient and public consultations (Research level); and Population consultations and Policy advisory groups (Policy‐making level).

Among the items that reached consensus, the mean score was 81.1% (SD = 5.29%), with a median of 78.6% (range: 75.0%–92.9%). Of the 92 items assessed in the first round, 24 reached consensus and 68 did not. The criterion most frequently associated with item consensus was equity (9/24), followed by significance (7/24), comprehensiveness (6/24) and adaptability (2/24). The criteria most frequently associated with lack of consensus were adaptability (21/68 items), comprehensiveness (17/68), significance (16/68) and equity (14/68). All scores for each criterion in the first round are presented in Table 2.

**Table 2:** Scores of strategies evaluated in Round 1 for the 4 consensus criteria.

Strategy name	Strategy score for each criterion (/100%), 1st round (14 participants, threshold: 75% agreement)			
	Equity^a^	Comprehensiveness^b^	Adaptability^c^	Significance^d^
Patient and public education	**76.9**	**75**	**76.9**	53.8
Behavioural change interventions	**76.9**	69.2	61.5	53.8
Personalized care planning	61.5	53.8	46.2	41.7
Self‐management supports	69.2	30.8	41.7	53.8
Shared decision making	61.5	61.5	53.8	69.2
Access to health records or portals	69.2	50	41.7	50
Patient and public navigation	**83.3**	**75**	66.7	58.3
Family supports	**75**	58.3	66.7	66.7
Peer supports	**83.3**	58.3	66.7	66.7
Information campaigns and platforms	71.4	57.1	64.3	50
Services users’ needs assessment	**85.7**	**85.7**	61.5	50
Quality safety assessment	57.1	46.2	50	50
Organizational advisory groups	**76.9**	**85.7**	**78.6**	**78.6**
Co‐leadership in quality and safety improvement	**85.7**	**85.7**	71.4	**92.9**
Patients and public as trainers	57.1	64.3	42.9	50
Co‐design of educational or training activities	**78.6**	64.3	57.1	**84.6**
Patient and public consultations	64.3	57.1	50	50
Involvement in study phases	69.2	71.4	57.1	**78.6**
Research advisory groups	64.3	71.4	57.1	**78.6**
Co‐leadership in research activities	57.1	71.4	42.9	**76.9**
Population consultations	69.2	71.4	50	53.8
Policy advisory groups	69.2	71.4	50	71.4
Co‐leadership in policymaking	69.2	**78.6**	50	**92.9**

*Note:* Bold values indicate those that reached the threshold.

^a^ **Equity:** Means that the strategy has the potential for involving patients and the public across diverse ethnic, sex, gender, health and socioeconomic backgrounds.

^b^ **Comprehensiveness:** Means that the strategy can be applied across all scaling phases, encompassing scalability assessment, the development of a scaling plan, preparation for scaling, implementation of the scaling plan and the evaluation and monitoring of the scaling process.

^c^ **Adaptability:** Means that the strategy could be adapted to different contexts, such as countries with different income levels, and to primary, secondary and tertiary levels of care.

^d^ **Significance:** Means that the strategy fosters the meaningful involvement of patients and the public as equal members of the scaling team.

#### 3.3.3 Second Round

By the end of Round 1, consensus had been achieved on one or more criteria for 22 of the PPI strategies and on all four criteria for one strategy. This left a total of 68 items distributed across 22 PPI strategies to be assessed in the second round. In this round, the mean consensus score was 69.4% (SD = 0.2%), with a median of 73.2% (range: 22.0%–100%). Thirteen PPI strategies reached the predefined consensus threshold of ≥ 80% on at least one of the four assessment criteria. Two strategies achieved full consensus across all four criteria, reinstating those that had not reached consensus in the first round: Service users' needs assessment reached consensus on adaptability and importance (88.9% for both), and Co‐leadership in quality and safety improvement reached consensus on adaptability (88.9%). Both strategies belong to the Organization of health and social care level.

Of the 22 PPI strategies assessed, seven reached consensus for equity, eight for comprehensiveness, three for adaptability, and one for significance. Policy advisory groups and co‐leadership in policymaking (both at the Policymaking level), as well as involvement in study phases and research advisory groups (both at the Research level), reached 100% consensus for equity. The latter two, together with patients and the public as trainees (Professional Training level), also reached 100% consensus for comprehensiveness. Four strategies did not reach consensus on any of the four criteria: Personalized care planning, Self‐management supports, Access to health records or portals (Direct care level) and Information campaigns and platforms (Organization of health and social care level).

For the criteria that reached consensus, the mean score was 92.4% (SD = 5.77%), with a median of 88.9% (range: 87.5%–100%). Of the 68 items assessed in the second round, 19 reached consensus and 49 did not. The criteria most frequently associated with consensus were comprehensiveness (8/19 items) and equity (7/19), followed by adaptability (3/19) and significance (1/19). The criteria most frequently associated with lack of consensus were importance (17/49 items), adaptability (16/49), equity (14/49) and comprehensiveness (13/49). All scores for each criterion in the second round are presented in Table 3.

**Table 3:** Scores of strategies evaluated in Round 2 for the 4 consensus criteria.

Strategy name	Strategy score for each criterion (/100%), 2nd round (9 participants, threshold: 80% agreement)			
	Equity^a^	Comprehensiveness^b^	Adaptability^c^	Significance^d^
Patient and public education	Not applicable^e^	Not applicable	Not applicable	71.4
Behavioural change interventions	Not applicable	**85.7**	75.0	37.5
Personalized care planning	62.5	37.5	37.5	37.5
Self‐management supports	62.5	37.5	50	42.9
Shared decision making	**87.5**	**87.5**	57.1	75
Access to health records or portals	66.7	22.2	22.2	44.4
Patient and public navigation	Not applicable	Not applicable	77.8	55.6
Family supports	Not applicable	55.6	44.4	55.6
Peer supports	Not applicable	77.8	77.8	66.7
Information campaigns and platforms	77.8	66.7	66.7	44.4
Services users’ needs assessment	Not applicable	Not applicable	**88.9**	**88.9**
Quality safety assessment	77.8	55.6	**88.9**	55.6
Organizational advisory groups	Not applicable	Not applicable	Not applicable	Not applicable
Co‐leadership in quality and safety improvement	Not applicable	Not applicable	**88.9**	Not applicable
Patients and public as trainers	75	**100**	66.7	77.8
Co‐design of educational or training activities	Not applicable	77.8	77.8	Not applicable
Patient and public consultations	**87.5**	62.5	75.0	50
Involvement in study phases	**100**	**100**	66.7	Not applicable
Research advisory groups	**100**	**100**	66.7	Not applicable
Co‐leadership in research activities	**88.9**	**88.9**	66.7	Not applicable
Population consultations	**88.9**	**88.9**	55.6	50
Policy advisory groups	**100**	**88.9**	75	56
Co‐leadership in policymaking	**100**	Not applicable	77.8	Not applicable

*Note:* Bold values indicate those that reached the threshold.

^a^ **Equity:** Means that the strategy has the potential for involving patients and the public across diverse ethnic, sex, gender, health, and socioeconomic backgrounds.

^b^ **Comprehensiveness:** Means that the strategy can be applied across all scaling phases, encompassing scalability assessment, the development of a scaling plan, preparation for scaling, implementation of the scaling plan, and the evaluation and monitoring of the scaling process.

^c^ **Adaptability:** Means that the strategy could be adapted to different contexts, such as countries with different income levels, and to primary, secondary, and tertiary levels of care.

^d^ **Significance:** Means that the strategy fosters the meaningful involvement of patients and the public as equal members of the scaling team.

^e^ **Not applicable:** Items that meet or exceed the 75% threshold for a given criterion in round 1 are excluded from round 2.

#### 3.3.4 Overall Synthesis

Out of the 23 PPI strategies assessed, 19 reached consensus on at least one of the four assessment criteria. Sixteen PPI strategies achieved consensus for equity, 14 for comprehensiveness, five for adaptability and eight for significance. Three strategies achieved consensus across all four criteria: Service users' needs assessment, Organizational advisory groups and Co‐leadership in quality and safety improvement.

The Service users' needs assessment strategy was recognized as a fundamental step in scaling interventions. However, one participant emphasized that this practice alone is not sufficient to ensure equity among stakeholders engaged in scaling processes. In contrast, collaborative governance strategies such as Organizational advisory groups and Co‐leadership in quality and safety improvement were perceived very positively. No negative comments were reported. Participant quotes illustrating these perceptions are presented in Box 2.

Box 2 **Illustrative participant quotes on perceptions of scaling strategies.**

**Table:** 

**Strategy**	**Participant quotes**
Service users’ needs assessment	Participant 5 (Trainee, Researcher and Member of a patient oriented research support institution): “*An important strategy, but not sufficient for becoming equal members of the scaling team.”*
Organizational advisory groups	Participant 2 (Patient and/or public representative): *“I dream of it… steering committees, participatory meetings and events, where the expertise of each partner in the ecosystem is used to analyze local needs”*
Co‐leadership in quality and safety	Participant 2 (Patient and/or public representative): *“Well, let's dream of sharing leadership and responsibility for initiatives aimed at improving quality and safety within the organization of health and social care services.”*

Four strategies (Personalized care planning, Self‐management supports, Access to health records or portals and Information campaigns and platforms) did not reach consensus on any criterion in either round. One participant explained that Personalized care planning and Self‐management supports are primarily individual‐level strategies and may not necessarily contribute to participatory processes in scaling. Regarding the PPI strategies Access to health records or portals and Information campaigns and platforms, participants acknowledged their importance for supporting basic levels of involvement (i.e., information sharing). However, they noted that literacy gaps and social inequalities continue to limit the effectiveness of these PPI strategies. Key participant quotes illustrating these perceptions are presented in Box 3.

Box 3 **Illustrative participant quotes on perceptions of scaling strategies.**

**Table:** 

**Strategies**	**Reason mentioned**
Personalized care planning and Self‐management supports	Participant 7 (Researcher): “*Advance care planning is not, in my opinion, a scaling strategy. It is a strategy to support the individual adoption of a practice. Likewise, self‐management is not, to me, a scaling strategy; it still represents an individual‐level change*.”
Self‐management supports and Information campaigns and platforms	Participant 5 (Trainee, Researcher and Member of a patient oriented research support institution): *“An action that is meant to promote equality can actually increase inequalities, for instance, a national campaign that does not specifically target disadvantaged populations would not enable optimal participation of all concerned.”*
	Participant 17 (Patient and/or public representative): *“This strategy may apply more effectively in privileged settings.”*

In terms of the global score across criteria, the highest‐ranked strategies were Co‐leadership in quality and safety improvement (88.3%), Co‐leadership in policymaking (87.3%) and Services users' needs assessment (87.3%). The strategies that achieved the highest consensus for equity were Co‐leadership in policymaking, Policy advisory groups, Research advisory groups and Involvement in study phases, all reaching 100% agreement.

However, participants highlighted equity challenges, emphasizing that the involvement of certain groups would require additional efforts and structural support. They noted that government and societal institutions play an essential role in ensuring equitable participation in decision‐making and policy processes. Regarding participation in research specifically, one participant emphasized that ensuring equity, diversity and inclusion (EDI) requires a proactive and adaptive approach. Key participant quotes illustrating these equity challenges are presented in Box 4.

Box 4 **Illustrative participant quotes on equity challenges in high‐scoring strategies.**

**Table:** 

Role of government and societal institutions	Participant 5 (Trainee, Researcher and Member of a patient oriented research support institution): *“There is a small caveat for people from rural and remote areas, for whom participation is more complex, creating a risk of disadvantage. Yet, this remains the most effective option.”*
	Participant 5 (Trainee, Researcher and Member of a patient oriented research support institution): *“Support from society and from political, governmental, and decision‐making bodies is crucial.”*
Proactive and adaptive approach	Participant 5 (Trainee, Researcher and Member of a patient oriented research support institution): *“As often, EDI requires a proactive approach. Some population groups will not be able to maintain long‐term engagement without significant accommodations.”*

Comprehensiveness, Patients and public as trainers, Research advisory groups and Involvement in study phases also reached 100% consensus, followed closely by Policy advisory groups and population consultations (88.9%). Overall, PPI strategies that target individuals rather than groups (e.g., self‐management supports and shared decision‐making) were viewed as interventions rather than as scaling strategies. One participant noted: “*Once again, there is confusion between the innovation itself and the strategy*.”

Regarding adaptability, the highest levels of consensus were observed for Quality safety assessment, Co‐leadership in quality and safety improvement and Services users' needs assessment, each with 88.9%. Some participants noted that the political and social context of different countries, not only their income level, can affect the effectiveness of PPI strategies. As one participant explained: “*I have some hesitation regarding countries with different income levels, and I would also add those with different types of governance (e.g., totalitarian regimes) or for instance, [differences in] the situation of women*.”

Finally, for significance, the top three strategies were Co‐leadership in policymaking and Co‐leadership in quality and safety improvement (both at 92.9%), followed by Services users' needs assessment (88.9%). Nevertheless, participants emphasized that simply informing or consulting patient and public representatives does not ensure genuine involvement. As one participant stated: “*The consultative aspect of committees is insufficient*.” Another added: “*Active involvement must be prioritized*.”

**Figure 3:**
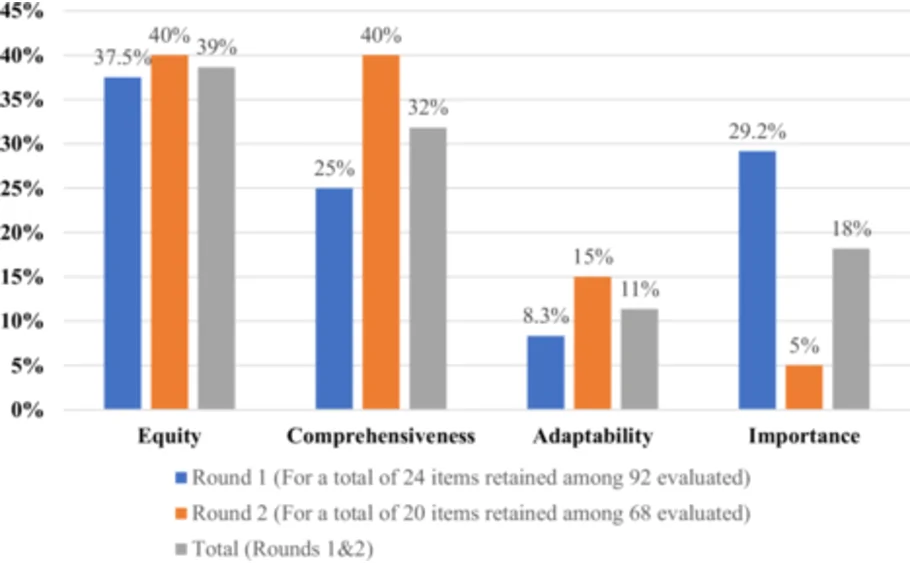
Evolution of consensus by evaluation criteria from one round to the next.

Out of the 92 items assessed, 44 reached consensus across both rounds, with equity being the most frequent (*n* = 17), followed by comprehensiveness (*n* = 14), significance (*n* = 8) and adaptability (*n* = 5). Figure 3 illustrates the evolution in the relative proportion of consensus achieved for each criterion across the two rounds of assessment.

#### 3.3.5 PPI Strategies in Scaling Across the System Levels

**Figure 4:**
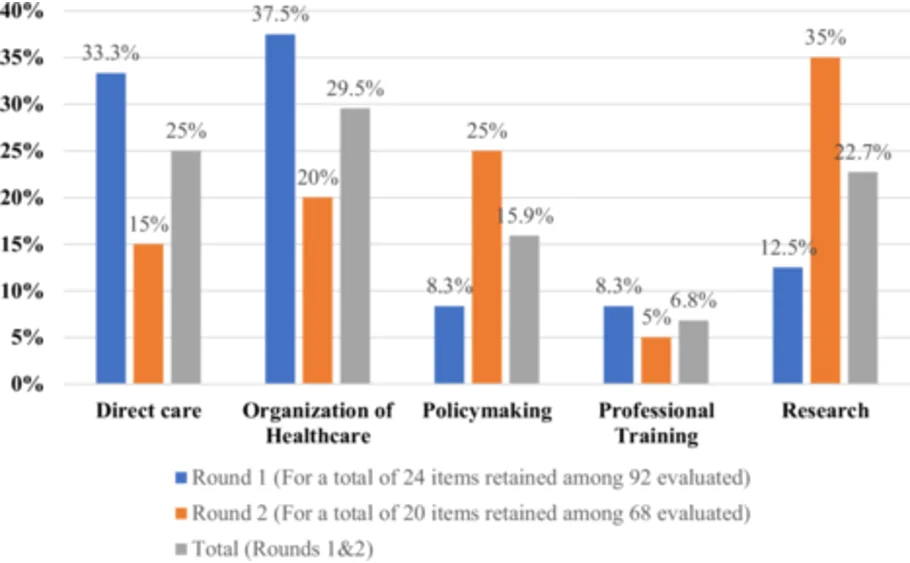
Distribution of criteria across system strata.

**Figure 5:**
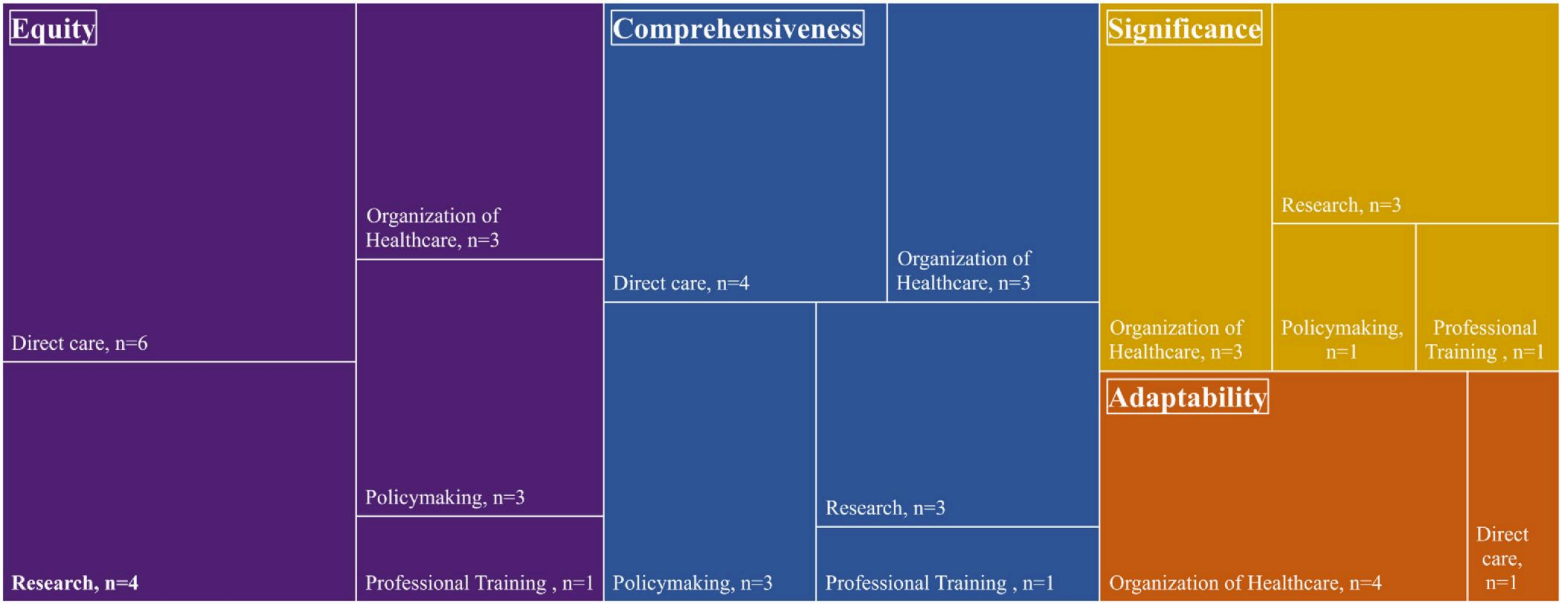
Frequency of associations between criteria and stratum of the healthcare system. Data labels indicate the number of strata associated with each criterion (total *n* = 44).

Most of the criteria that reached consensus were associated with PPI strategies in scaling situated within the Organization of health and social care (*n* = 13) system level. This was followed by Direct Care (*n* = 11), Research (*n* = 10), Policymaking (*n* = 7) and Professional Training (*n* = 3). Figure 4 illustrates the distribution of criteria across system strata. Sixteen associations between assessment criteria and system levels were identified (Figure 5). The most frequently observed associations were Equity—Direct Care (6 out of 44 items), followed by Equity—Research (4/44), Adaptability— Organization of health and social care level (4/44) and Comprehensiveness—Direct Care (4/44).

#### 3.3.6 PPI Strategies in the Involvement Continuum

All 44 criteria that reached consensus were relevant across the four levels of the patient and public involvement continuum (information, consultation, collaboration and co‐production). Six criteria apply to information, six to consultation, two to collaboration and nine to co‐production. These associations are not mutually exclusive, as some items may correspond to more than one level.

## 4 Discussion

We conducted a participatory consensus study inspired by Delphi techniques. Patient and public representatives were involved alongside researchers, health professionals and policymakers to assess whether 23 strategies identified in the literature were relevant for promoting equity, comprehensiveness, adaptability and significance throughout PPI in scaling. Three strategies achieved consensus across all four criteria. Four strategies did not reach consensus on any criterion. Among all criteria assessed, consensus was most frequently reached for equity and least frequently for adaptability. Overall, the criteria that reached consensus were relevant for all four levels of the patient and public involvement continuum, with the highest frequency observed in co‐production [8, 27, 28, 29]. These findings led to the following observations.

First, our study highlights important gaps between the strategies that scaling stakeholders identified as most effective for promoting equity, comprehensiveness, adaptability and significance in PPI, and the strategies most frequently used in real‐world scaling interventions [8]. Service users' needs assessment, Organizational advisory groups and Co‐leadership in quality and safety improvement were consistently perceived as relevant across all four criteria. Notably, Co‐leadership in quality and safety improvement achieved the highest global score. However, these strategies were among the least frequently reported in a 2024 scoping review of current PPI scaling practices [8]. This discrepancy suggests that scaling efforts may be overlooking strategies with the strongest potential to ensure meaningful patient and public involvement. Based on our findings, we strongly recommend that scaling initiatives to improve the quality and safety of health and social care prioritize approaches that engage patient and public representatives through shared leadership and responsibility, for example by actively including community members in quality improvement teams [25].

Second, considering the PPI strategies that did not achieve consensus for any criterion (Personalized care planning, Self‐management supports, Access to health records or portals and Information campaigns and platforms), most were developed for the Direct Care level and, within the PPI continuum, were situated at the information level [8]. In particular, these strategies did not reach consensus for significance, indicating that they were not perceived as fosteringgenuine and meaningful involvement of patients and the public as equal members of the scaling team. Information is a prerequisite for PPI, but our findings raise the question of whether it should be regarded as an ultimate goal of PPI in scaling, since it does not ensure genuine involvement in the same way as other forms of engagement.

Third, according to scaling stakeholders, the vast majority of PPI strategies assessed could enable scaling teams to engage patients and the public from diverse ethnic, sex, gender, health and socioeconomic backgrounds, thereby fostering equity in scaling. This is a highly valuable result, since advancing equity is increasingly recognized as a central aim of the science of scaling [1, 5, 44]. Much debate has focused on both the limitations and the potential of existing scaling frameworks and tools (e.g., scalability tools) to adequately address this aim [5, 44]. Considering equity as a goal in PPI in scaling aligns with an ethical perspective in which the involvement of patient and public representatives is regarded as the right of every potential user of health and social services, rather than merely as a means of increasing the effectiveness of scaling interventions [8, 44]. However, we found in our study that adapting PPI strategies to different scaling contexts can be a challenge, confirming previous consensus studies that highlighted adaptability as a key concept for scaling and underscoring the need for further research on this topic [20].

Finally, consensus was reached on criteria that spanned the whole PPI continuum (information, consultation, collaboration and co‐production), with the highest frequency of consensus observed in co‐production [8]. Again, this finding reflects a disconnect with the strategies most often mobilized in real‐world conditions. In our study, Co‐leadership in quality and safety improvement and Co‐leadership in policymaking were among the strategies with the highest global scores, yet they were reported infrequently in the 2024 scoping review [8]. Based on these results, we strongly encourage scaling teams to engage with patient and public representatives from the very beginning of scaling projects and throughout all scaling phases, as recommended by scaling frameworks [3, 45].

## 5 Limitations

A limitation of our study was the geographical concentration of participants, with most being based in Canada. However, this was partly mitigated by the grounding of our work in a 2024 scoping review that did not exclude evidence on the basis of country of origin. Furthermore, the composition of our research team, which included scaling experts in diverse practitioner, policy and researcher roles from low and high‐income countries, ensured that the study benefited from a broad range of international perspectives and contextual expertise. Although we recruited two fewer participants than initially expected, all planned categories of participants were represented in the research. Two former steering committee members whose institutional roles changed during the study subsequently participated in the consenus panel. However, we consider their prior involvement to have limited influence on the findings, as the steering committee and the consensus panel served distinctive purposes. The main limitation occurred at the level of survey completion, as some individuals who had consented did not complete both rounds of the online consultation. This reflects a common challenge in online consensus processes, where time constraints, digital access, and competing professional or personal priorities may limit full participation. Nevertheless, the diversity of perspectives captured across stakeholder categories supports the robustness of our findings.

## 6 Conclusion

We conducted a participatory consensus study to identify the most relevant strategies for meaningful PPI in scaling, that is, those with the potential to involve patients and the public in scaling in an equitable, comprehensive, adaptable and significant way. Our findings demonstrate that while numerous strategies are available for involving patients and the public in scaling health and social care interventions, those considered most effective by stakeholders are not always the ones most frequently mobilized in practice. Our participants found that most strategies promoted equity, confirming its central role in the science and ethics of scaling. In contrast, adaptability emerged as a persistent challenge and requires further research. Consensus was reached regarding strategies applied at all levels of the PPI continuum, with the highest frequency observed in co‐production. This reinforces the importance of shared leadership and responsibility in scaling. Based on these results, we recommend that scaling teams systematically involve patient and public representatives from the outset and across all phases of scaling. Priority should be given to approaches that strengthen equity and accountability, as reflected in current international frameworks.

## Author Contributions

**Roberta de Carvalho Corôa:** conceptualization, methodology, validation, visualization, writing—original draft, writing—review and editing. **Odilon Quentin Assan:** writing—original draft, methodology, data curation, formal analysis, visualization, writing—review and editing. **Ali Ben Charif:** conceptualization, investigation, funding acquisition, writing—review and editing, validation, project administration, supervision, resources. **Amédé Gogovor:** writing—review and editing, validation, conceptualization, funding acquisition. **Kathy Kastner:** conceptualization, funding acquisition, validation, writing—review and editing. **Robert K. D. McLean:** conceptualization, funding acquisition, writing—review and editing, validation. **Andrew Milat:** conceptualization, funding acquisition, validation, writing—review and editing. **France Légaré:** conceptualization, investigation, funding acquisition, writing—original draft, methodology, validation, visualization, writing—review and editing, project administration, supervision, data curation, resources. **The RePOS Network:** conceptualization, validation, visualization.

Our project is funded by a Catalyst Grant (#PAO‐169411) from the Canadian Institutes of Health Research (CIHR). Roberta de Carvalho Corôa was funded by the Postdoctoral Training Scholarships (Citizens of other countries) by the Fonds de recherche en santé du Québec–Santé (FRQ‐S). France Légaré holds a Tier 1 Canada Research Chair in Shared Decision Making and Knowledge Mobilization. The funding agreement ensures the authors' independence in designing the study, writing and publishing this article. The information provided or views expressed in this article are the responsibility of the authors alone.

## Ethics Statement

This study received approval from the Research Ethics Board of the Centre Intégré Universitaire de Santé et de Services Sociaux de la Capitale‐Nationale (Approval No. 2021‐2019). All participants provided informed consent prior to participation.

## Conflicts of Interest

The authors declare no conflicts of interest.

## Data Availability

The data generated and analyzed during this study are not publicly available because they contain information that could compromise participant confidentiality. De‐identified data may be made available from the corresponding author upon reasonable request and subject to applicable ethical and institutional requirements.
